# Bodyprint—A Meta-Feature Based LSTM Hashing Model for Person Re-Identification

**DOI:** 10.3390/s20185365

**Published:** 2020-09-18

**Authors:** Danilo Avola, Luigi Cinque, Alessio Fagioli, Gian Luca Foresti, Daniele Pannone, Claudio Piciarelli

**Affiliations:** 1 Department of Computer Science, Sapienza University, 00198 Rome, Italy; cinque@di.uniroma1.it (L.C.); fagioli@di.uniroma1.it (A.F.); pannone@di.uniroma1.it (D.P.); 2 Department of Communication and Social Research, Sapienza University, 00198 Rome, Italy; 3 Department of Mathematics, Computer Science and Physics, University of Udine, 33100 Udine, Italy; gianluca.foresti@uniud.it

**Keywords:** person re-identification, long short-term memory networks, 2D skeletons, RGB video sequences, joints based meta-features, binary coding, hashing

## Abstract

Person re-identification is concerned with matching people across disjointed camera views at different places and different time instants. This task results of great interest in computer vision, especially in video surveillance applications where the re-identification and tracking of persons are required on uncontrolled crowded spaces and after long time periods. The latter aspects are responsible for most of the current unsolved problems of person re-identification, in fact, the presence of many people in a location as well as the passing of hours or days give arise to important visual appearance changes of people, for example, clothes, lighting, and occlusions; thus making person re-identification a very hard task. In this paper, for the first time in the state-of-the-art, a meta-feature based Long Short-Term Memory (LSTM) hashing model for person re-identification is presented. Starting from 2D skeletons extracted from RGB video streams, the proposed method computes a set of novel meta-features based on movement, gait, and bone proportions. These features are analysed by a network composed of a single LSTM layer and two dense layers. The first layer is used to create a pattern of the person’s identity, then, the seconds are used to generate a bodyprint hash through binary coding. The effectiveness of the proposed method is tested on three challenging datasets, that is, iLIDS-VID, PRID 2011, and MARS. In particular, the reported results show that the proposed method, which is not based on visual appearance of people, is fully competitive with respect to other methods based on visual features. In addition, thanks to its skeleton model abstraction, the method results to be a concrete contribute to address open problems, such as long-term re-identification and severe illumination changes, which tend to heavily influence the visual appearance of persons.

## 1. Introduction

Last years have seen the design of increasingly advanced computer vision algorithms to support a wide range of critical tasks in a plethora of application areas. These algorithms often have the responsibility of taking determining decisions in issues where failure would lead to serious consequences. In References [1,2,3], for example, vision-based systems are used for inspection of pipeline infrastructures. In particular, in the first work, the authors presented a method for subsea pipeline corrosion estimation by using colour information of corroded pipes. To manage the degraded underwater images, the authors developed ad-hoc pre-processing algorithms for image restoration and enhancement. Differently, in the second and third work, the authors focused on large infrastructures, such as sewers and waterworks. Both works proposed an anomaly detector based on unsupervised machine learning techniques, which, unlike other competitors in this specific field, do not require annotated samples for training the detection models. Correct evaluations by the algorithms reported above can provide countless advantages in terms of maintenance and hazard determination. Even in hand and body rehabilitation, as shown in References [4,5,6], last few years have seen the proliferation of vision-based systems able to provide measurements and predictions about the recovery degree of lost skills by patients affected with strokes and degenerative diseases. The first work focused on a hand skeleton model together with pose estimation and tracking algorithms both to determine palm and fingers pose estimation and to track their movements during a rehabilitation exercise. Similarly, but using a body skeleton model, the second and third work used a customized long short term memory (LSTM) model to analyze movements and limitations of patients’ body, treating a full body rehabilitation exercise like a long action over time. Hand and body skeleton models, in addition to allowing the transposition of patients’ virtual avatars into immersive environments, also allow therapists to better detect joints that require of more exercises, thus optimizing and customizing the recovery task.

Without doubt, one of the application areas in which computer vision has had more impact, in the last ten years, is the active video surveillance, that is, those automatic systems able to replace human operators in complex tasks, such as intrusion detection [7,8], event recognition [9,10], target identification [11,12], and many others. In References [13], for example, the authors proposed a reinforcement learning approach to train a deep neural network to find optimal patrolling strategies for Unmanned Aerial Vehicles (UAVs) visual coverage tasks. Unlike other works in this application area, their method explicitly considers different coverage requirements expressed as relevance maps. References [14,15,16], instead, even if use pixel-based computer vision techniques, that is, low level processing, are able to achieve remarkable results in terms of novelty recognition and change detection, thus providing a step forward in the field of aerial monitoring. Moving to stationary and Pan–Tilt–Zoom (PTZ) cameras, very recent works, as that reported in Reference [17], shown that, even in challenging application fields, robust systems can be implemented and applied, for security contexts, in everyday life. In particular, the authors proposed a continuous-learning framework for context-aware activity recognition from unlabelled video. In their innovative approach, the authors formulated an information-theoretic active learning technique that utilizes contextual information among activities and objects. Other interesting current examples are presented in References [18,19]. In the first, a novel end-to-end partially supervised deep learning approach for video anomaly detection and localization using only normal samples is presented. Instead, in the second, the authors propose an abnormal event detection hybrid modulation method via feature expectation subgraph calibrating classification in video surveillance scenes. Both works proven to provide remarkable results in terms of robustness and accuracy.

Active surveillance systems, in real contexts, are composed of different algorithms, each of which collaborates with the others for the achievement of a specific target. For example, algorithms for separating background from foreground, for example, References [20,21,22], are often used, as pre-processing stage, both to detect the objects of interest in the scene and to have a reference model of the background and its variations over time. Another example are the tracking algorithms, for example, Reference [10,23], which are used to analyze moving objects. Among these collaborative algorithms, person re-identification ones, for example, References [24,25], play a key role, especially in security, protection, and prevention areas. In fact, being able to identify a person’s identity and being able to verify the presence of that person in other locations hours or even weeks before is a fundamental step for the areas reported above. Anyway, despite the efforts of many computer vision researchers in this application area, person re-identification task still presents several problems largely unsolved. Most of the person re-identification methods, in fact, are based on visual features extracted from images to model a person’s appearance [26,27]. This leads to a first class of problems since, as known, visual features have many weaknesses, including illumination changes, shadows, direction of light, and many others. Another class of problems regards the background clutter [28,29] and occlusions [30,31], which tend, in uncontrolled environments, to lower system performances in term of accuracy. A final class of problems, very important from a practical point of view, is referred to the long-term re-identification and camouflage [32,33]. Many systems, based totally or in part on visual features, are not able to re-identify persons under the two issues reported above, thus making the use of these systems limited in real contexts.

In this paper, a meta-feature based LSTM hashing model for person re-identification is presented. The proposed method takes inspiration from some of our recent experiences in using 2D/3D skeleton based features and LSTM models to recognize hand gestures [34], body actions [35], and body affects [36] in long video sequences. Unlike these, the 2D skeleton model, in this paper, is used to generate biometric features referred to movement, gait, and bone proportions of the body. Compared to the current literature, beyond the originality of the overall pipeline and features, the proposed method presents several novelties suitably studied to address, at least in part, the classes of problems reported above, especially in uncontrolled and crowded environments. First, the method is fully based on features extracted from 2D skeleton models present in a scene. The algorithm used to extract the models, reported in Reference [37], is proven to be highly reliable in terms of accuracy, even for multi-person estimation. The algorithm uses each input image only to produce the 2D locations of anatomical keypoints for each person in the image. This means that all the aspects, for example, illumination changes, direction of light, and many others, that can influence the re-identification by systems based on visual appearance of people, can, in the proposed method, be overlooked since the images are only used to generate 2D skeletons. Second, the proposed pipeline does not use visual features, but only features derived by analysing the 2D skeleton joints. These features, through the use of the LSTM model, are designed to catch both dynamic correlations of different body parts during movements and gaits as well as bone proportions of relevant body parts. The term meta-features was born from the fact that our features are hence conceived to generalize the body in terms of movements, gaits, and proportions. Thanks to this abstraction, long-term re-identification and some kinds of camouflages can be better managed. Third, thanks to the two final layers of the proposed network, which implement the bodyprint hash through binary coding, the representative space of our method can be considered uncountable, thus providing a tool to potentially label each and every human being in the world. The proposed method was tested on three challenging datasets designed for person-re-identification in video sequences, that is, iLIDS-VID [38], Person Re-ID (PRID) 2011 [39], and Motion Analysis and Re-identification Set (MARS) [40], showing remarkable results, compared with key works of the current literature, in terms of re-identification rank. Summarizing, the contributions of the paper with respect to both the present open issues and the current state-of-the-art in terms of pipelines and models can be outlined as follows:

- Concerning the present person re-identification field open issues, the proposed method, which is not based on visual appearance, can be considered a concrete support in uncontrolled crowded spaces, especially in long-term re-identification where people, usually, change clothes and some aspects of their visual appearance over time. In addition, the use of RGB cameras allows the method to be used in both indoor and outdoor environments; overcoming, thanks to the use of 2D skeletons, well-known problems related to scene analysis, including illumination changes, shadows, direction of light, background clutter, and occlusions.
- Regarding the overall pipeline and model, the approach proposed in this paper presents different novelties. First, even if some features are inspired by selected works of the current literature in recognizing hand gestures, body actions, and body affects; other features are completely new as also is their joint utilize. Second, for the first time in the literature, an LSTM hashing model for person re-identification is used. This model was conceived not only to exploit the dynamic patterns of the body movements, but also to provide, via the last two layers, a mechanism by which to provide a labelling approach for millions of people thanks to binary coding properties.

The rest of the paper is structured as follows. In Section 2, a concise literature review focused on person re-identification methods based on video sequences processing is presented. In Section 3, the entire methodology is described, including the proposed LSTM hashing model and meta-features. In Section 4, three benchmark datasets and comparative results with other literature works, are reported. Finally, Section 5 concludes the paper.

## 2. Related Work

In this section, selected works that treat person re-identification task on the basis of video sequences by deep learning techniques are presented and discussed. The same works are then reported in the experimental tests, that is, Section 4, for a full comparison.

A first remarkable work is reported in Reference [40], where the authors, first and foremost, introduced the Motion Analysis and Re-identification Set (MARS) dataset, a very large collection of challenging video sequences that, moreover, were also used for part of the experimental tests in the present paper. Beyond the dataset, the authors also reported an extensive evaluation of the state-of-the-art methods, including a customized one based on Convolutional Neural Networks (CNNs). The latter, supported by the Cross-view Quadratic Discriminant Analysis (XQDA) metric learning scheme [41] on iLIDS-VID and PRID 2011 datasets, and by Multiple Query (MQ), only on the MARS dataset, shown to outperform several competitive approaches, thus demonstrating a good generalization ability. The work proposed in Reference [42], instead, adopts a Recurrent Neural Network (RNN) architecture in which features are extracted from each frame by using a CNN. The latter incorporates a recurrent final layer that allows information to flow between time-steps. To provide an overall appearance feature for the complete sequence, the features, from all time-steps, are then combined by using temporal pooling. The authors conduced experiments on iLIDS-VID and PRID-2011 datasets, obtaining, on both, very significant results. Other two competitors, of the work proposed in this paper, are described in References [43,44]. In the first, the authors presented an end-to-end deep neural network architecture, which integrates a temporal attention model to selectively focus on the discriminative frames and a spatial recurrent model to exploit the contextual information when measuring the similarity. In the second, the authors described a deep architecture with jointly attentive spatial-temporal pooling, enabling a joint learning of the representations of the inputs as well as their similarity measurement. Their method extends the standard RNN-CNNs by decomposing pooling into two steps—a spatial-pooling on feature map from CNN and an attentive temporal-pooling on the output of RNN. Both works were tested on iLIDS-VID, PRID 2011, and MARS, obtaining outstanding results compared with different works of the current literature. The authors of Reference [45] proposed a method for extracting a global representation of subjects through the several frames composing a video. In particular, the method attends human body part appearance and motion simultaneously and aggregates the extracted features via the vector of locally aggregated descriptors (VLAD) [46] aggregator. By considering the adversarial learning approach, in Reference [47] the authors presented a deep few-shot adversarial learning to produce effective video representations for video-based person re-identification, using few labelled training paired videos. In detail, the method is based on Variational Recurrent Neural Networks (VRNNs) [48], which can capture temporal dynamics by mapping video sequences into latent variables. Another approach is to consider the walking cycle of the subjects, such as the one presented in Reference [49], where the authors proposed a super-pixel tracking method for extracting motion information, used to select the best walking cycle through an unsupervised method. A last competitor is reported in Reference [50], where a deep attention based Siamese model to jointly learn spatio-temporal expressive video representations and similarity metrics is presented. In their approach, the authors embed visual attention into convolutional activations from local regions to dynamically encode spatial context priors and capture the relevant patterns for the propagation through the network. Also this work was compared on the three mentioned benchmark datasets, showing remarkable results.

## 3. Methodology: LSTM Hashing Model and Meta-Features

**Figure 1:**
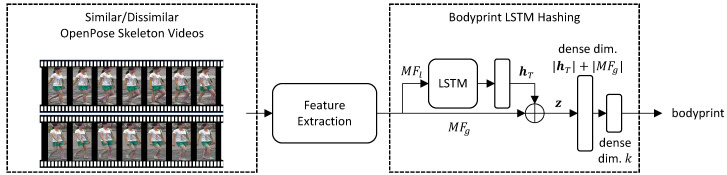
Long short term memory (LSTM) bodyprint hashing architecture scheme.

In this section, the proposed architecture for person re-identification, based on skeleton meta-features derived from RGB videos and bodyprint LSTM hashing, is presented. A scheme summarizing the architecture is shown in Figure 1. In detail, starting from an RGB video, the OpenPose [37] library is first used to obtain skeleton joints positions. This representation is then fed to the feature extraction module, where meta-features analysing movements both locally (i.e., in a frame-by-frame fashion) and globally (e.g., averaging over the whole motion) are generated, fully characterizing a person. Subsequently, meta-features are given as input to the bodyprint LSTM hashing module. In this last component, local meta-features are first analysed via a single LSTM layer. The LSTM representation of local meta-features is then concatenated to global meta-features and finally fed to two dense layers, so that a bodyprint hash is generated through binary coding.

### 3.1. Skeleton Joint Generation

**Figure 2:**
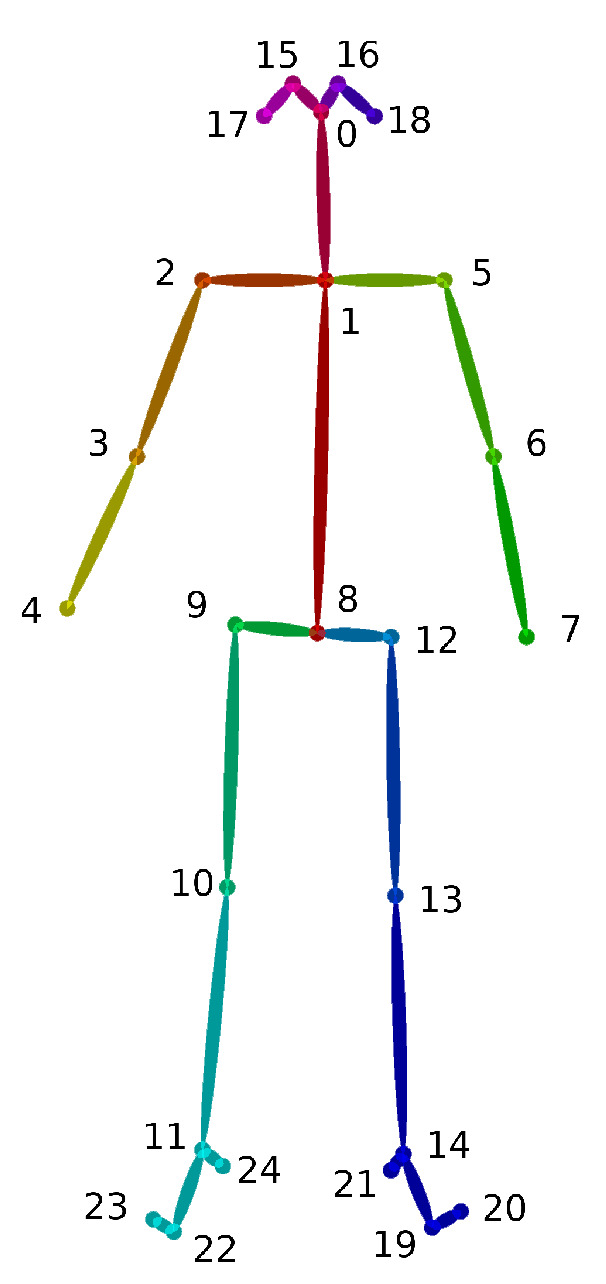
OpenPose body-foot keypoint schematic.

The first and foremost step, for a video-based person re-identification system using skeleton information, is a skeleton joint generation phase. At this stage, the well-known OpenPose library is exploited so that the main joints of a human skeleton can be extracted from RGB video sequences. In detail, this library leverages a multi-stage CNN so that limbs Part Affinity Fields (PAFs), a set of 2D vector fields encoding location and orientation of limbs over the image domain, are generated. By exploiting these fields, OpenPose is able to produce accurate skeletons from RGB frames and track them inside RGB videos. The extensive list of skeleton joints, representing body-foot position estimations, is depicted in Figure 2. As can be seen, up to 25 joints are described for a skeleton, where a joint is defined by its (x,y) coordinates inside the RGB frame. The identified joints correspond to—nose (0), neck (1), right/left shoulder, elbow, wrist (2–7), hips middle point (8), right/left hip, knee, ankle, eye, ear, big toe, small toes, and heel (9–24). While joint positions alone do not provide useful information, due to their strict correlation to the video they are extracted from, they can still be used to generate a detailed description of body movements, via the feature extraction module.

### 3.2. Feature Extraction

In this module, skeleton joint positions identified via OpenPose are used to generate a detailed description of full-body movements. Skeleton joint positions can be exploited to produce meaningful features able to describe, for example, gait, using the lower half of the body as shown by several gait re-identification works [51,52], or body emotions, leveraging features extracted from both lower and upper body halves [36]. Motivated by the encouraging results already obtained via hand-crafted skeleton features in References [34,35,36], two meta-feature groups built from skeleton joint positions are proposed in this work to analyze the whole body: local and global meta-features defined $MF_{l}$ and $MF_{g}$, respectively.

Concerning the $MF_{l}$ set, a frame-by-frame analysis of body movements is provided via the upper and lower body openness, frontal and sideways head tilt, lower, upper, head, and composite body wobble, left and right limbs relative positions, cross-limb distance and ratio, head, arms, and legs location of activity, as well as lower, upper, and full-body convex triangulation meta-features, for a total of 21 local descriptors per frame. Through these features, gait cues and relevant changes associated to the entire body structure can be captured as they happen.

Regarding the $MF_{g}$ group, a condensed description of body characteristics and movements is specified via the head, arms, and legs relative movement, limp, as well as feet, legs, chest, arms, and head bones extension meta-features, for a total of 19 global descriptors. Through these features, bias towards specific body parts, which might become apparent during a walk, and detailed body conformations, describing possible limb length discrepancy, for example, can be depicted.

#### 3.2.1. Body Openness

Body Openness (BO) is employed as devised in a previous study [36]. This meta-feature can describe both lower and upper body openness, defined $BO_{l}$ and $BO_{u}$, respectively. The former, is computed using the ratio between the ankles-hips distance, and left/right knees distance; indicating whether a person has an open lower body (i.e., open legs) or has bent legs. Similarly, $BO_{u}$ is calculated utilising the ratio between left/right elbows distance, and the neck-hips distance, thus capturing a broaden out chest. Intuitively, low $BO_{l}$ values depict bent legs, corresponding to a crouched position; mid-range $BO_{l}$ values correspond to straight yet open legs; while high $BO_{l}$ values denote straight but closed legs (i.e., standing position). Comparably, low $BO_{u}$ values indicate a broaden out chest; mid-range $BO_{u}$ values correspond to straight arms and torso; while high $BO_{u}$ values depict open elbows and bent torso. Formally, given a video sequence *S*, these quantities are computed for each frame f∈S as follows:

(1) $$BO_{l}=\frac{d(hip_{middle},\;ankles_{avg})}{d(knee_{left},\;knee_{right})},$$

(2) $$BO_{u}=\frac{d(neck,\;hip_{middle})}{d(elbow_{left},\;elbow_{right})},$$

where d(·,·) is the Euclidean distance; $hip_{middle}$, $knee_{left}$, $knee_{right}$, neck, $elbow_{left}$, and $elbow_{right}$, correspond to OpenPose joints 8, 13, 10, 1, 6, and 3, respectively; while $ankles_{avg}$ indicates the average (x,y) ankles position, that is, OpenPose joints 11 and 14. Summarizing, BO is a local meta-feature (i.e., $BO\in MF_{l}$) describing 2 quantities, that is, lower and upper body half openness.

#### 3.2.2. Head Tilt

Head Tilt (HT) reports the degree of frontal and sideways head tilt, defined $HT_{fr}$ and $HT_{s}$, respectively. The former, is calculated exploiting the angle between the neck-hips and nose-neck axes. The latter, is computed using the angle between the nose-neck and left-right eye axes. Intuitively, for positive $HT_{fr}$ values, the head is facing downward, while for negative values the head is facing upward. Similarly, for positive $HT_{s}$ values the head is tilted to the right side, while for negative values there is a tilt to the left side. Formally, given a video sequence *S*, these measures are calculated for each frame f∈S as follows:

(3) $$HT_{fr}=\frac{m_{nose-neck}-m_{neck-hip_{middle}}}{1+m_{neck-hip_{middle}}*m_{nose-neck}},$$

(4) $$HT_{s}=\frac{m_{eye_{left}-eye_{right}}-m_{nose-neck}}{1+m_{nose-neck}*m_{eye_{left}-eye_{right}}},$$

where nose, neck, $hip_{middle}$, $eye_{left}$, and $eye_{right}$, represent the OpenPose joints 0, 1, 8, 16, 15, respectively; while the slope *m* of a given axis is computed using:

(5) $$m=\tan\theta =\frac{y_{2}-y_{1}}{x_{2}-x_{1}},$$

where *x* and *y* indicate the joint coordinates used to compute the various axes. Summarizing, HT is a local meta-feature (i.e., $HT\in MF_{l}$) indicating 2 measures, that is, frontal and sideways head tilt.

#### 3.2.3. Body Wobble

Body Wobble (BW) describes whether a person has an unsteady head, upper or lower body part, defined $BW_{h}$, $BW_{u}$ and $BW_{l}$ measures, respectively. Moreover, this meta-feature is employed to also indicate the composite wobbling degree of a person, designated as $BW_{c}$, by accounting for the head, upper and lower halves of the body when computing this quantity. Similarly to HT, the angles between the neck-hips axis and either the left-right eye, shoulder, or hip axes, are exploited to compute these values. Moreover, the composite body wobble is computed by averaging the absolute values of head, upper and lower body wobble, to depict the general degree of body wobble. Intuitively, $BW_{h}$, $BW_{u}$ and $BW_{l}$ describe toward which direction the corresponding body part is wobbling during a motion, while $BW_{c}$ characterizes the movement by capturing possible peculiar walks (e.g., a drunk person usually wobbles more than a sober one). Formally, given a video sequence *S*, $BW_{h}$, $BW_{u}$ and $BW_{l}$ are computed for each frame f∈S as follows:

(6) $$BW_{h}=\frac{m_{eye_{left}-eye_{right}}-m_{neck-hip_{middle}}}{1+m_{neck-hip_{middle}}*m_{eye_{left}-eye_{right}}},$$

(7) $$BW_{u}=\frac{m_{shoulder_{left}-shoulder_{right}}-m_{neck-hip_{middle}}}{1+m_{neck-hip_{middle}}*m_{shoulder_{left}-shoulder_{right}}},$$

(8) $$BW_{l}=\frac{m_{hip_{left}-hip_{right}}-m_{neck-hip_{middle}}}{1+m_{neck-hip_{middle}}*m_{hip_{left}-hip_{right}}},$$

where $eye_{left}$, $eye_{right}$, neck, $hip_{middle}$, $shoulder_{left}$, $shoulder_{right}$, $hip_{left}$, and $hip_{right}$, indicate the OpenPose joints 16, 15, 1, 8, 5, 2, 12, and 9, respectively. Finally, the composite body wobble $BW_{c}$ is derived by the other body wobble meta-features as follows:

(9) $$BW_{c}=\frac{|BW_{h}|+|BW_{u}|+|BW_{l}|}{3}.$$

Summarizing, BW is a local meta-feature (i.e., $BW\in MF_{l}$) denoting 4 values, that is, lower, upper, head, and composite body wobble.

#### 3.2.4. Limbs Relative Position

Limbs Relative Position (LRP) indicates the opposing arm and leg relative position with respect to the neck-hips axis (i.e., a vertical axis). This meta-feature is defined for left arm/right leg and right arm/left leg pairs, named $LRP_{lr}$ and $LRP_{rl}$, respectively. Intuitively, an arm and the opposing leg tend to be synchronised when walking, and oscillate together. Thus, by computing the difference between opposing limbs and the vertical neck-hips axis (i.e., their relative position), it is possible to describe whether the synchronous oscillation is happening or not. Formally, given a video sequence *S*, relative positions are computed for each frame f∈S as follows:

(10) $$\begin{array}{cc} LRP_{lr} & =\Delta relative\;distance(neck,hips,arm_{left_{avg}},leg_{right_{avg}})\\ \; & =distance\left(neck,hips,arm_{left_{avg}}\right)-distance\left(neck,hips,leg_{right_{avg}}\right)\\ \; & =\frac{|\left(neck_{y}-hips_{y}\right)arm_{left_{avg_{x}}}-\left(neck_{x}-hips_{x}\right)arm_{left_{avg_{y}}}+neck_{x}hips_{y}-neck_{y}hips_{x}|}{\sqrt{{\left(neck_{y}-hips_{y}\right)}^{2}+{\left(neck_{x}-hips_{x}\right)}^{2}}}\\ \; & -\frac{|\left(neck_{y}-hips_{y}\right)leg_{right_{avg_{x}}}-\left(neck_{x}-hips_{x}\right)leg_{right_{avg_{y}}}+neck_{x}hips_{y}-neck_{y}hips_{x}|}{\sqrt{{\left(neck_{y}-hips_{y}\right)}^{2}+{\left(neck_{x}-hips_{x}\right)}^{2}}}, \end{array}$$

(11) $$\begin{array}{cc} LRP_{rl} & =\Delta relative\;distance(neck,hips,arm_{right_{avg}},leg_{left_{avg}})\\ \; & =distance\left(neck,hips,arm_{right_{avg}}\right)-distance\left(neck,hips,leg_{left_{avg}}\right)\\ \; & =\frac{|\left(neck_{y}-hips_{y}\right)arm_{right_{avg_{x}}}-\left(neck_{x}-hips_{x}\right)arm_{right_{avg_{y}}}+neck_{x}hips_{y}-neck_{y}hips_{x}|}{\sqrt{{\left(neck_{y}-hips_{y}\right)}^{2}+{\left(neck_{x}-hips_{x}\right)}^{2}}}\\ \; & -\frac{|\left(neck_{y}-hips_{y}\right)leg_{left_{avg_{x}}}-\left(neck_{x}-hips_{x}\right)leg_{left_{avg_{y}}}+neck_{x}hips_{y}-neck_{y}hips_{x}|}{\sqrt{{\left(neck_{y}-hips_{y}\right)}^{2}+{\left(neck_{x}-hips_{x}\right)}^{2}}}, \end{array}$$

where neck and hips correspond to OpenPose joints 1, and 8, respectively; while $arm_{left_{avg}}$, $arm_{right_{avg}}$, $leg_{left_{avg}}$, and $leg_{right_{avg}}$ are the average (x,y) positions of left arm, right arm, left leg, and right leg, computed using joints (5, 6, 7), (2, 3, 4), (12, 13, 14), and (9, 10, 11), respectively. Summarizing, LRP is a local meta-feature (i.e., $LRP\in MF_{l}$) depicting 2 measures, that is, left/right and right/left arm-leg relative position.

#### 3.2.5. Cross-Limb Distance and Ratio

Cross-Limb Distance Ratio (CLDR) denotes the cross distance between left arm/right leg and right arm/left leg pairs, as well as their ratio, defined $CLDR_{lr}$, $CLDR_{rl}$, and $CLDR_{r}$, respectively. Similarly to the LRP meta-feature, CLDR represents the synchronised oscillation of opposite limbs, although using only the average limbs position and their ratio instead of a reference axis. Intuitively, low $CLDR_{rl}$, and $CLDR_{rl}$ distances indicate a synchronous cross-limb oscillation; while high values denote an irregular movement. Concerning $CLDR_{r}$, the closer its value is to 1, the more synchronised is the oscillation. Indeed, through these meta-features, possible peculiar movements can be grasped during a motion. For example, arms held behind the back while walking would result in low synchronous oscillation, and could be captured via low $CLDR_{lr}$, $CLDR_{rl}$, and $CLDR_{r}$ values. Formally, given a video sequence *S*, cross-limb distances are computed for each frame f∈S as follows:

(12) $$CLDR_{lr}=d\left(arm_{left_{avg}},\;leg_{right_{avg}}\right),$$

(13) $$CLDR_{rl}=d\left(arm_{right_{avg}},\;leg_{left_{avg}}\right),$$

where d(·,·) is the Euclidean distance; while $arm_{left_{avg}}$, $arm_{left_{avg}}$, $leg_{left_{avg}}$, and $leg_{left_{avg}}$ are the average (x,y) positions of left arm, right arm, left leg, and right leg, computed using joints (5, 6, 7), (2, 3, 4), (12, 13, 14), and (9, 10, 11), respectively. Finally, $CLDR_{r}$, that is, the cross-limb distance ratio, is calculated using the following equation:

(14) $$CLDR_{r}=\frac{1}{1+CLDR_{lr}-CLDR_{rl}}.$$

Summarizing, CLDR is a local meta-feature (i.e., $CLDR\in MF_{l}$) describing 3 values, that is, left/right, right/left arm-leg cross limb relative position as well as their ratio.

#### 3.2.6. Location of Activity

Location of Activity (LOA) quantifies movement of each body component, and is computed for the head, left/right arms and legs, defined $LOA_{h}$, $LOA_{al}$, $LOA_{ar}$, $LOA_{ll}$, and $LOA_{lr}$, respectively. Intuitively, these meta-features analyse two consecutive frames to capture position changes, and directly measure the movement of a given component, so that they can determine which part is being moved the most during the motion. For example, a person with arms flailing about, would result in high $LOA_{al}$ and $LOA_{ar}$ values. Formally, given a video sequence *S*, the set of components to be analysed C={h,al,ar,ll,lr}, and the set containing all joints of a given body part $J_{c},c\in C$, the various $LOC_{c}$ are computed for each pair of consecutive frames $f,f^{\prime }\in S$ as follows:

(15) $$LOA_{c,f,f^{\prime }}=\Delta c_{f,f^{\prime }}=\sum_{j\in J_{c}}\frac{d(j_{f},\;j_{f^{\prime }})}{|J_{c}|},$$

where c∈C={h,al,ar,ll,lr} and h,al,ar,ll,lr correspond to head, $arm_{left}$, $arm_{right}$, $leg_{left}$, and $leg_{right}$ components, composed by joints (0, 1, 15, 16, 17, 18), (5, 6, 7), (2, 3, 4), (12, 13, 14), and (9, 10, 11), respectively; while d(·,·) is the Euclidean distance. Summarizing, LOA is a local meta-feature (i.e., $LOA\in MF_{l}$) computing 5 values, that is, head, arms, and legs location of activity.

#### 3.2.7. Body Convex Triangulation

Body Convex Triangulation (BCT) depicts the center of mass distribution by analysing triangle structures built between neck and wrists joints; middle hips and ankles joints; as well as head and feet joints. These meta-features, defined $BCT_{u}$, $BCT_{l}$, and $BCT_{f}$, represent the upper, lower, and full-body mass distribution, respectively. Intuitively, by analysing the triangle inner angles ratio difference, it is possible to describe whether a person is leaning left, right, or has a balanced mass distribution (i.e., positive, negative, and 0 values, respectively) in relation to the upper, lower, and full-body triangles observed during a motion. Formally, given a triangle with vertices *A*, *B*, and *C*, the corresponding angles $\theta_{\alpha },\theta_{\beta },\theta_{\gamma }$ are first computed as follows:

(16) $$\theta_{\alpha }=\cos^{-1}\left(\frac{a\cdot b}{∥a∥∥b∥}\right),$$

(17) $$\theta_{\beta }=\cos^{-1}\left(\frac{c\cdot d}{∥c∥∥d∥}\right),$$

(18) $$\theta_{\gamma }=180-\theta_{\alpha }-\theta_{\beta },$$

where · is the dot product; ∥·∥ represents the vector magnitude; while a, b, c, and d, indicate vectors $\vec{AB}$, $\vec{AC}$, $\vec{BA}$, and $\vec{BC}$, respectively. Then, given a video sequence *S*, the difference between the ratios of angles adjacent to the triangle base, and the non adjacent one, is used to compute the $BCT_{l}$, $BCT_{u}$, and $BCT_{f}$ values for each frame f∈S, via the following equations:

(19) $$BCT_{l}=\frac{\theta_{ankle_{left}}}{\theta_{hips_{middle}}}-\frac{\theta_{ankle_{right}}}{\theta_{hips_{middle}}},$$

(20) $$BCT_{u}=\frac{\theta_{wrist_{left}}}{\theta_{neck}}-\frac{wrist_{right}}{\theta_{neck}},$$

(21) $$BCT_{f}=\frac{\theta_{heel_{left}}}{\theta_{nose}}-\frac{heel_{right}}{\theta_{nose}},$$

where $ankle_{left}$, $ankle_{right}$, and $hips_{middle}$, that is, OpenPose joints 14, 11, 8, correspond to the lower-body triangle; $wrist_{left}$, $wrist_{right}$, and neck, that is, joints 7, 4, 1, denote the upper-body triangle; while $heel_{left}$, $heel_{right}$, and nose, that is, joints 21, 24, 0, indicate the full-body triangle. Summarizing, BCT is a local meta-feature (i.e., $BCT\in MF_{l}$) denoting 3 quantities, that is, lower, upper, and full-body convex triangulation.

#### 3.2.8. Relative Movement

Relative Movement (RM) describes the amount of change a given body part has with respect to the whole body, as conceived in Reference [36]. This meta feature is computed for the head, left/right arm, and left/right leg, defined $RM_{h}$, $RM_{al}$, $RM_{ar}$, $RM_{ll}$, and $RM_{lr}$, respectively. Intuitively, RM first analyses position and velocity changes for each body component, then computes the ratio between a single body part position (or velocity) variation amount, and the sum of position (or velocity) changes for all body components. Formally, given a video sequence *S*, the set of components to be analysed C={h,al,ar,ll,lr}, and the set containing all joints of a given body part $J_{c},c\in C$, the average change of a component ($AC_{c}$), over the entire recording *S*, is computed as follows:

(22) $$AC_{c}=\frac{\sum_{f=0}^{|S|-2}\sum_{j\in J_{c}}\frac{\left|\Delta (j_{f},\;j_{f+1})\right|}{|J_{c}|}}{|S|-2},$$

where c∈C={h,al,ar,ll,lr} and *h*, al, ar, ll, lr, correspond to head, $arm_{left}$, $arm_{right}$, $leg_{left}$, and $leg_{right}$ components, composed by joints (0, 1, 15, 16, 17, 18), (5, 6, 7), (2, 3, 4), (12, 13, 14), and (9, 10, 11), respectively. Finally, the $RM_{c},\;c\in C$, is derived using the following equation:

(23) $$RM_{c}=\frac{AC_{c}}{\sum_{c\in C}AC_{c}}.$$

Summarizing, RM is a global meta-feature (i.e., $RM\in MF_{g}$) computed for both position and velocity changes of head, arms, and legs; thus resulting in 10 distinct values describing the entire recording *S*.

#### 3.2.9. Limp

Limp (*L*) denotes whether or not a leg is moved less than the other one when walking. This meta feature is calculated using the average velocity difference between left and right legs, over the entire video sequence. Intuitively, a limping person generally has much lower velocity in either leg. Thus, this aspect can be captured by exploiting *L*, where a limp in the right or left leg is denoted via a negative or positive *L* value. Formally, given a video sequence *S*, the Limp *L* is computed as follows:

(24) $$L=\sum_{f=0}^{|S|-2}\sum_{j\in J_{leg_{left}}}\frac{\left|v(j)\right|}{|S|-2}-\sum_{f=0}^{|S|-2}\sum_{j^{\prime }\in J_{leg_{right}}}\frac{\left|v(j^{\prime })\right|}{|S|-2},$$

where $J_{leg_{left}}$ and $J_{leg_{right}}$ represent the joint sets of left and right leg, defined by OpenPose joints (12, 13, 14) and (9, 10, 11), respectively; while v(·) is the joint velocity which can be easily computed using position variations and video frame per second (FPS). Summarizing, *L* is a single value global meta-feature (i.e., $L\in MF_{g}$) indicating limp over the whole video sequence *S*.

#### 3.2.10. Bones Extension

Bones Extension (BE) describes left/right foot, left/right leg, chest, left/right arm, and head bones extension, defined $BE_{fl}$, $BE_{fr}$, $BE_{ll}$, $BE_{lr}$, $BE_{ct}$, $BE_{al}$, $BE_{ar}$, and $BE_{h}$ respectively. Intuitively, these meta-features provide a bone size estimation by exploiting the maximum distance between the two end-points of a bone, over the entire video sequence. Formally, given a video sequence *S*, the set of limb bones *B* and the sets of joints describing the bones $J_{b},b\in B$; $BE_{b}$ is computed as follows:

(25) $$BE_{b}=\sum_{\begin{array}{c} j,j^{\prime }\in J_{b}\\ s.t\;j∼j^{\prime } \end{array}}\max_{f\in S}d\left(j,j^{\prime }\right),$$

where d(·,·) is the Euclidean distance; ∼ identifies adjacent joints of a given bone; while $BE_{fl}$, $BE_{fr}$, $BE_{ll}$, $BE_{lr}$, $BE_{ct}$, $BE_{al}$, $BE_{ar}$, and $BE_{h}$ denote the left foot, right foot, left leg, right leg, chest, left arm, right arm, and head, via OpenPose joint sets (19, 21), (22, 24), (12, 13, 14), (9, 10, 11), (1, 8), (5, 6, 7), (2, 3, 4), (0, 1), respectively. Summarizing, BE is global meta-feature (i.e., $BE\in MF_{g}$) defining bone length over feet, legs, chest, arms, and head, for a total of 8 distinct values over the entire recording *S*.

### 3.3. Bodyprint LSTM Hashing

The last step of the proposed framework, is the construction of bodyprints through the binary coding technique. In this last module, the set $MF_{l}$ is analysed through a LSTM so that time variations of local meta-features can be fully captured. The LSTM output is then concatenated to the set $MF_{g}$, so that time-invariant body characteristics are merged together with time-varying ones. Finally, two dense layers are employed to implement the binary coding technique, so that a unique representation for a given person is built, ultimately allowing re-identification of that person.

#### 3.3.1. LSTM

All local meta-features $MF_{l}$ are generated for each frame of the input video sequence containing skeleton joint positions. While the proposed features provide a detailed local description of the motion (i.e., for each specific frame), they do to not account for possible time correlation between two different frames of the input sequence. To fully exploit this information, a single layer LSTM network was chosen due to its inherent ability to handle input sequences [53]. This network leverages forget gates and peep-hole connections so that non relevant information is gradually ignored, thus improving its ability to correlate both close and distant information in a given sequence. Formally, a generic LSTM cell at time *t* is described by the following equations:

(26) $$i_{t}=\sigma \left(W_{xi}x_{t}+W_{hi}h_{t-1}+W_{ci}c_{t-1}+b_{i}\right),$$

(27) $$f_{t}=\sigma \left(W_{xf}x_{t}+W_{hf}h_{t-1}+W_{cf}c_{t-1}+b_{f}\right),$$

(28) $$c_{t}=f_{t}c_{t-1}+i_{t}\tanh\left(W_{xc}x_{t}+W_{hc}h_{t-1}+Wb_{c}\right),$$

(29) $$o_{t}=\sigma \left(W_{xo}x_{t}+W_{ho}h_{t-1}+W_{co}c_{t}+b_{o}\right),$$

(30) $$W_{t}=\sigma_{t}\tanh\left(c_{t}\right),$$

where i, f, o, c, and h represent input gate, forget gate, output gate, cell activation, and hidden vectors, respectively. Moreover, $W_{xi}$, $W_{xf}$, $W_{xo}$, and $W_{xc}$ are weights connecting the various components to the input, while $W_{ci}$, $W_{cf}$, and $W_{co}$ correspond to diagonal weights for peep-hole connections. Additionally $b_{i}$, $b_{f}$, $b_{o}$, and $b_{c}$ denote the bias associated to input gate, forget gate, output gate, and cell. Finally, the Hadamard product is used for vector multiplication. To conclude, the LSTM output $h_{T}$, that is, the last hidden state summarizing motion characteristics of a *T*-length video sequence, is concatenated to $MF_{g}$, consequently producing a z vector of size $|h_{T}|+|MF_{g}|$, representing a body motion.

#### 3.3.2. Bodyprint Hashing

The body motion characterization z can be transformed into a unique identifier via binary coding hashing, so that a bodyprint is ultimately built and used for person re-identification. Following the approach in Reference [54], Supervised Deep Hashing (SDH) with a relaxed loss function is exploited to obtain a bodyprint binary code, produced by feeding the z-vector to two dense layers. The first layer is used to merge the concatenation of global and local meta-features, while the second one is used to obtain a binary code of dimension *k*. Intuitively, through SDH, similar body descriptions should result in similar codes and vice-versa, ultimately enabling a bodyprint to be used in re-identification since it is associated to a specific person.

The key aspect of this SDH approach is the relaxed loss function, where the Euclidean distance between the hash of two samples is used in conjunction with a regularizer to relax the binary constraint. This relaxation is necessary due to the sign function, usually used to obtain binary codes, leading to a discontinuous, non-differentiable and non-treatable problem via back-propagation. Formally, given two input sequences $S_{1}$ and $S_{2}$ and their corresponding bodyprints $b_{1},b_{2}\in {\left\{-1,+1\right\}}^{k}$, it is possible to define y=0 in case the sequences are similar (i.e., they are derived from the same person), and y=1 otherwise. Consequently, the relaxed loss $L_{r}$ with respect to the two sequences is computed as follows:

(31) $$\begin{array}{cc} L_{r}\left(b_{1},b_{2},y\right) & =\frac{1}{2}\left(1-y\right){∥b_{1}-b_{2}∥}_{2}^{2}\\ \; & +\frac{1}{2}y\max(m-∥b_{1}-b_{2}{∥}_{2}^{2},\;0)\\ \; & +\alpha (∥|b_{1}|-1{∥}_{1}+∥|b_{2}|-1{∥}_{1}), \end{array}$$

where ${∥\cdot ∥}_{1}$ and ${∥\cdot ∥}_{2}$ represent L1 and L2-norm, respectively; 1 denotes an all-ones vector; |·| indicates the element-wise absolute value operation; m>0 is a similarity threshold; while α is a parameter modulating the regularizer weight. In detail, the first term penalises sequences generated from the same person and mapped to different bodyprints; the second term punishes sequences of different persons encoded to close binary codes, according to the threshold *m*; while the third term is the regularizer exploited to relax the binary constraint. Supposing there are *N* pairs randomly selected from the training sequences $\left\{S_{i,1},S_{i,2},y_{i}|i=1,\ldots ,N\right\}$, the resulting loss function to be minimised is:

(32) $$\begin{array}{cc} L_{r} & =\sum_{i=1}^{N}\{\frac{1}{2}\left(1-y_{i}\right){∥b_{i,1}-b_{i,2}∥}_{2}^{2}\\ \; & +\frac{1}{2}y_{i}\max(m-∥b_{i,1}-b_{i,2}{∥}_{2}^{2},\;0)\\ \; & +\alpha (∥|b_{i,1}|-1{∥}_{1}+∥|b_{i,2}|-1{∥}_{1}\},\\ s.t.\;b_{i,j} & \in {\left\{-1,+1\right\}}^{k},\;i\in \left\{1,\ldots ,N\right\},\;j\in \left\{1,2\right\}. \end{array}$$

While this function can be applied via the back-propagation algorithm with mini-batch gradient descent method, the subgradients of both max and absolute value operations are non-differentiable at certain points. Thus, as described in Reference [54], the partial derivatives at those points are defined to be 1 and are computed, for the three terms of Equation (32) $T_{1},T_{2},T_{3}$, as follows:

(33) $$\begin{array}{cc} \frac{\partial T_{1}}{\partial b_{i,j}} & ={\left(-1\right)}^{j+1}\left(1-y_{i}\right)\left(b_{i,1}-b_{1,2}\right).\\ \frac{\partial T_{2}}{\partial b_{i,j}} & =\left\{\begin{array}{cc} {\left(-1\right)}^{j}y_{i}\left(b_{i,1}-b_{i,2}\right), & ∥b_{i,1}-b_{i,2}{∥}_{2}^{2}<m;\\ 0, & otherwise. \end{array}\right.\\ \frac{\partial T_{3}}{\partial b_{i,j}} & =\alpha \delta \left(b_{i,j}\right),\;\delta \left(x\right)=\left\{\begin{array}{cc} 1, & -1\leq x\leq 0\;or\;x\geq 1;\\ -1, & otherwise. \end{array}\right. \end{array}$$

To conclude, bodyprints can be computed by applying sign(b), and this final representation is then exploited for person re-identification as extensively shown in the experimental section.

## 4. Results and Discussion

In this section, the results obtained with the proposed method are discussed and compared with other state-of-the-art approaches. The comparisons are performed on three public video re-identification datasets, which are discussed below.

### 4.1. Datasets and Settings

Concerning the datasets, the experiments were performed on iLIDS-VID [38], PRID 2011 [39], and MARS [40]. The iLIDS-VID dataset is comprised of 600 image sequences belonging to 300 people acquired by two non-overlapping cameras. Each sequence has a number of frame ranging from 23 to 192, with an average of 73. The PRID 2011 dataset consists of 400 image sequences belonging to 200 people acquired by two adjacent cameras. Each sequence has a number of frame ranging from 5 to 675, with an average of 100. Finally, the MARS dataset consists of 1261 videos acquired by 2 to 6 cameras. The entire dataset contains 20,175 tracklets, of which 3248 are distractors. The chosen datasets are challenging for the following reasons. iLIDS-VID presents clothing similarities, occlusions, cluttered background and variations across camera views. PRID 2011 main challenges are the lighting and, as for iLIDS-VID, the variations across camera views. MARS, instead, has the above mentioned challenges besides the distractors.

**Figure 3:**
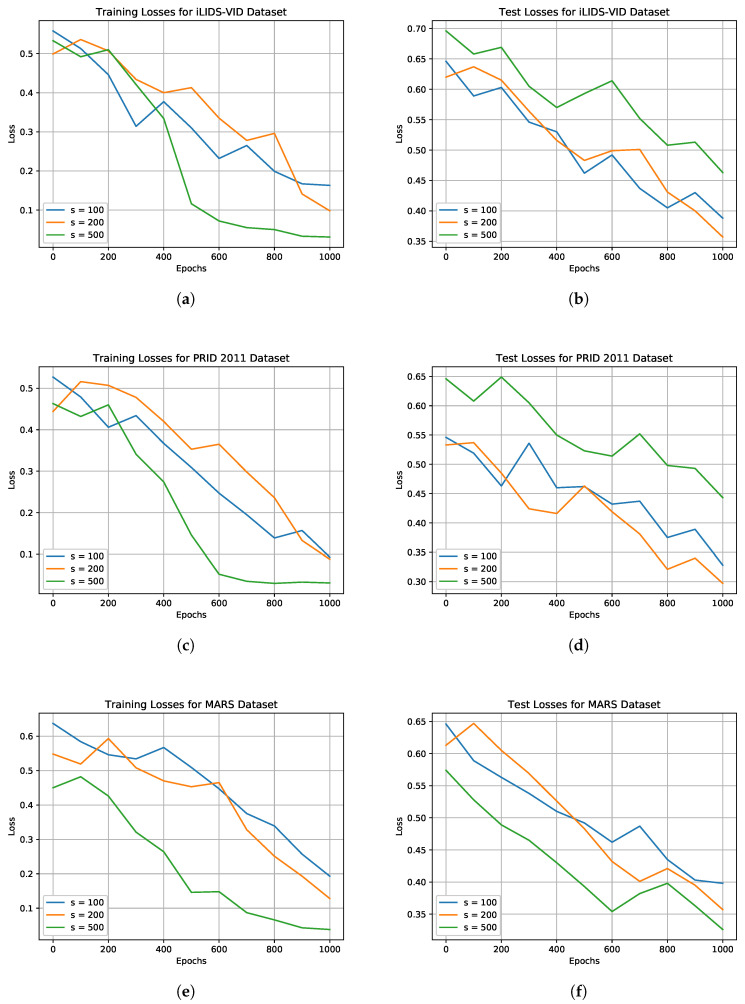
Train (left column) and test (right column) losses of the proposed method obtained on: iLIDS-VID, PRID 2011, and MARS datasets. Performances for these three datasets are summarized in (**a**,**b**), (**c**,**d**), and (**e**,**f**), respectively. By increasing the LSTM representational power (i.e., *s* size) convergence on the training set is reached much faster (i.e., (**a**,**c**,**e**) figures). However, due to the low number of identities in iLIDS-VID and PRID 2011 datasets, the highest *s* amount (i.e., 500) might result in overfitting scenarios (i.e., (**b**,**d**) figures).

Concerning the model, it was implemented in Pytorch and the following parameters were used. For the LSTM input, a tensor having a shape of [256,50,21] was employed, where 256 is the batch size, 50 are the frames used for each subject and 21 are the local meta-features $MF_{l}$. For the LSTM output a tensor having a shape of [256,s] is instead utilized, where s∈[100,200,500]. The value of *s* was chosen empirically during the training of the model. As depicted in Figure 3, for iLIDS-VID and PRID 2011, that is, the dataset with the smaller number of identities, we used 200 as value for *s* to have better results over s=100 and to avoid the overfitting, obtained with s=500. Instead, for MARS dataset *s* was set to 500 due to the high number of identities. A value of *s* higher than 500 led to a high training time with a negligible accuracy improvement. In relation to the dense layers, the first one had an input size of $s+|MF_{g}|$, namely 119, 219, and 519, while the second dense layer used a dimension k∈[16,32,64] to create the bodyprint representation. Regarding the tensor precision, since the used meta-features comprise ratios, the tensor data type was set as a 32-bit float number. For the relaxation parameter α and similarity threshold *m*, 0.01 and 2 were chosen, respectively. The model was trained on a NVidia RTX 2080 GPU for 1000 epochs with a learning rate of 0.001.

### 4.2. Experiment Analysis

In Table 1, the results obtained with the proposed method are compared with current key works of the state-of-the-art. In particular, the comparison was performed with deep network based methods, namely, RNN [42], CNN + XQDA + MQ [40], Spatial and Temporal RNN (SPRNN) [43], Attentive Spatial-Temporal Pooling Network (ASTPN) [44], Deep Siamese Network (DSAN) [50], PersonVLAD + XQDA [45], VRNN + KissME [47], and Superpixel-Based Temporally Aligned Representation (STAR) [49]. Regarding the proposed method, 5 different versions were used for comparison. The *Bodyprint_local_* uses only local features, *Bodyprint_global_* uses only global features, while *Bodyprint_k_*, k∈[16,32,64], uses both local and global features but with different size of the bodyprint hashing. By first analysing the local-only and global-only version of bodyprint, it is possible to observe that for the iLIDS-VID dataset performances are consistent with the state-of-the-art. For the PRID 2011 and MARS datasets, instead, there is a noticeable drop in the performance. This can be associated with the higher number of identities and to the fact that the proposed model was designed to use synergistically local and global features. In general, we have that the local-only version of the model performs better with respect to the global-only. This is amenable to the fact that due their granularity, the local features have a better description power, while the global features can result similar for different subjects. By considering, instead, the full bodyprint model, we have that starting from the 16-bits size hashing vector, the obtained ranking can overcome many state-of-the-art works.

Concerning the iLIDS-VID and the state-of-the-art algorithm that has the best rank-1 performance on it, that is, PersonVLAD, we have that Bodyprint_16_ performs 2.8% worst with respect to it. However, with higher bodyprint vector sizes, that is, 32 and 64 bits, there are an in line performance between Bodyprint_32_ and PersonVLAD, and a 2.7% improvement by using the 64 bits bodyprint version. Moreover, by considering the second best algorithm, that is, ASTPN, the gain obtained with 32 and 64 bits bodyprint vectors is 8.3% and 11.4%, respectively, which is an impressive result. For the PRID 2011 dataset, we have that Bodyprint_16_ and Bodyprint_32_ rank-1 results are in line with the other methods. In detail, Bodyprint_16_ is 4% below SPRNN (i.e., the second best performing rank-1 method on this dataset) while Bodyprint_32_ is 0.1% over it. For rank-5 and rank-20, we have that both Bodyprint_16_ and Bodyprint_32_ are slightly below SPRNN results. Regarding Bodyprint_64_, we have that it is the best algorithm at rank-5, while it is the third best result for rank-1 and rank-20, in which PersonVLAD has the best results. Finally, considering the MARS dataset, we have that at rank-1 Bodyprint_16_ and Bodyprint_32_ are in line with the state-of-the-art, while Bodyprint_64_ substantially outperforms other literature works. Conversely, for the rank-5, we have that Bodyprint_64_ is the second best method after PersonVLAD, which has obtained a score of 94.9%. For the rank-20, instead, Bodyprint_64_ is in line with the other works, by obtaining a value of 95.3%. Despite the method performing generally well, there are some limitations that can influence the accuracy. These limitations are discussed in the next section.

### 4.3. Limitations

The proposed model presents two major limitations: occlusions and static frames (i.e., only one frame per subject). These two situations strongly influence the feature and meta-feature extraction and computation, leading to a worse performance of the model. Regarding the occlusions, we have that for the global features the average value is lowered with respect to the number of the occluded frames. For the local features, instead, we have two cases. The first case occurs when the lower part of a subject is occluded, hence only the upper local features are available. In this case, 9 local features are available. On the contrary, for the second case we have that the upper part of the subject is occluded, allowing the extraction of the lower local features only. In this case, 5 local features are available. Despite in the first case there are 4 more local features, the performance of both cases are almost identical. Since the proposed model has been designed to consider the whole body of a subject, we have that some global features cannot be computed in case of occlusions, contributing to the lowering of the performance. Concerning the static frames, we have that all the meta-features that need more than one frame to be computer are set to 0. This means that those features lose their descriptive potential and, as for the occlusions, there is a drop in the performance. In detail, when used with static frames or in sequences with a lot of occlusions the rank-1 value is around 55%.

A final remark must be made on the quality of the analysed images. Since the proposed method strongly relies on OpenPose framework, an important requirement is that the frames containing the subjects must not be too small in terms of spatial resolution. Otherwise, OpenPose will not extract all the skeleton parts, leading to the same problems encountered with occlusions. The same does not hold for data acquired with depth cameras, since the skeleton is directly provided and not estimated from RGB images. Anyway, considering the very good results of the proposed system and considering also that it is not based on visual features, thus overcoming a wide range of drawbacks of these systems, we can conclude that the system proposed in this paper can be considered a real contribute to the scientific community about this topic.

## 5. Conclusions

In this paper, a novel meta-feature based LSTM hashing model for person re-identification in RGB video sequences is presented. The proposed method is not based on visual features, but on meta-features extracted from the 2D skeleton models present in the scene. The meta-features are designed to catch movements, gaits, and bone proportions of a human body, thus providing an abstraction useful to overcome a wide range of drawbacks of the common competitors, including long-term re-identification and camouflage. The usefulness of the proposed method was tested on three benchmark dataset, that is, iLIDS-VID, PRID 2011, and MARS; thus demonstrating a step forward in the current literature.

## Figures and Tables

**Table 1:** Quantitative comparison between the proposed method and the current state-of-the-art methods on the chosen datasets. The best results are highlighted in bold.

Method	iLIDS-VID			PRID 2011			MARS		
	Rank-1	Rank-5	Rank-20	Rank-1	Rank-5	Rank-20	Rank-1	Rank-5	Rank-20
RNN [42]	58	84	96	70	90	97	-	-	-
CNN + XQDA + MQ [40]	53	81.4	95.1	77.3	93.5	99.3	68.3	82.6	89.4
SPRNN [43]	55.2	86.5	97	79.4	94.4	99.3	70.6	90	97.6
ASTPN [44]	62	86	94	77	95	99	44	70	81
DSAN [50]	61.9	86.8	98.6	77	96.4	99.4	73.5	85	97.5
PersonVLAD + XQDA [45]	70.7	88.2	**99.2**	**88**	96.2	**99.7**	82.8	**94.9**	**99**
VRNN + KissME [47]	64.6	90.2	97.9	84.2	96.9	98.9	61.2	79.5	96.9
STAR [49]	67.5	91.7	98.8	69.2	94.9	99.1	80	89.3	95.1
Bodyprint_local_	58.7	80.1	92.1	67.4	85.3	96.5	55.7	67	72.3
Bodyprint_global_	56	78.4	90.6	65.9	83.2	95.5	55.4	64.9	72.2
Bodyprint_16_	67.9	88.5	94.3	75.4	90.8	98	76.5	84.4	90.2
Bodyprint_32_	70.3	90.1	95.6	79.5	92.3	98.7	77	87.4	93.9
Bodyprint_64_	**73.4**	**94.2**	99.1	82.7	**97**	99.2	**86.5**	92.6	95.3
